# Hypoxia-dependent recruitment of error-prone DNA polymerases to genome replication

**DOI:** 10.1038/s41388-024-03192-0

**Published:** 2024-10-28

**Authors:** Ran Yehuda, Ido Dromi, Yishai Levin, Thomas Carell, Nicholas Geacintov, Zvi Livneh

**Affiliations:** 1 Dept. of Biomolecular Sciences, Weizmann Institute of Science, Rehovot, 7610001 Israel; 2 The de Botton Institute for Protein Profiling of the Nancy and Stephen Grand Israel National Center for Personalized Medicine, Weizmann Institute of Science, Rehovot, 7610001 Israel; 3 Center for Integrated Protein Science at the Department of Chemistry, Ludwig-Maximilians- Universität, München, Butenandtstrasse 5-13, 81377 München, Germany; 4 Chemistry Department, New York University, New York, NY USA

**Keywords:** Biological sciences, Genetics

## Abstract

Hypoxia is common in tumors and is associated with cancer progression and drug resistance, driven, at least in part, by genetic instability. Little is known on how hypoxia affects Translesion DNA Synthesis (TLS), in which error-prone DNA polymerases bypass lesions, thereby maintaining DNA continuity at the price of increased mutations. Here we show that under acute hypoxia, PCNA monoubiquitination, a key step in TLS, and expression of error-prone DNA polymerases increased under regulation of the HIF1α transcription factor. Knocking-down expression of DNA polymerase η, or using PCNA ubiquitination-resistant cells, inhibited genomic DNA replication specifically under hypoxia, and iPOND analysis revealed massive recruitment of TLS DNA polymerases to nascent DNA under hypoxia, uncovering a dramatic involvement of error-prone DNA polymerases in genomic replication. Of note, expression of TLS-polymerases correlates with *VEGFA* (primary HIF1α target) in a database of renal cell carcinoma, a cancer which accumulates HIF1α. Our results suggest that the tumor microenvironment can lead the cell to forgo, to some extent, the fast and accurate canonical DNA polymerases, for the more flexible and robust, but low-fidelity TLS DNA polymerases. This might endow cancer cells with resilience to overcome replication stress, and mutability to escape the immune system and chemotherapeutic drugs.

## Introduction

Hypoxia, a hallmark of tumor microenvironment, is common in solid tumors and is associated with invasion, metastasis and drug resistance [1]. The hypoxic condition of the tumor is due to its rapid growth, which outgrows the surrounding vascular system [2]. The lack of oxygen induces in the cancer cell expression of angiogenesis factors such as VEGF, leading to new blood vessels formation in the tumor, consequently leading to its further rapid growth and, hence the reoccurrence of hypoxic conditions. This hypoxia–reoxygenation cycle induces reactive oxygen species (ROS) and the DNA damage response (DDR) pathway [3, 4]. Yet, although DDR is increased under hypoxia, essentially all the DNA repair mechanisms tested are suppressed under hypoxic conditions [5–9]. A key regulator induced by hypoxia is HIF1, a transcription factor which fulfills a major role in the cell’s adaptation to hypoxia, such as inducing angiogenesis, glycolysis, and more [10]. The protein is a heterodimer in which the HIF1α subunit is degraded under normoxic conditions via oxygen-dependent proline hydroxylation by prolyl hydroxylases (PHD), which allows recognition by the VHL E3 ligase, leading to polyubiquitination and degradation of the protein [11]. Under hypoxia PHD cannot add hydroxyl groups to HIF1α and therefore VHL cannot recognize it, leading to HIF1α accumulation. Thus, HIF1α acts as a limiting factor for the transcription factor HIF1 [11].

Little is known about the effect of hypoxia on translesion DNA synthesis (TLS), a DNA damage tolerance mechanism in which unrepaired DNA lesions are bypassed by specialized error-prone DNA polymerases [12, 13]. This can occur at or behind the replication fork, enabling the continuity of DNA at the price of increased mutations, formed due to the miscoding nature of DNA lesions and the low fidelity of TLS polymerases [14, 15]. Since TLS is both mutagenic and assists replication progression, two functions that facilitate cancer development, we hypothesized that TLS might be recruited to the malignant process. Here we report, that hypoxia, acting via the HIF1 pathway, facilitates PCNA monoubiquitination, and induces several key TLS DNA polymerases which are massively recruited to nascent genomic DNA, and participate in genome replication under hypoxia.

## Results

### Hypoxia induces PCNA monoubiquitination and expression of TLS DNA polymerases

**Fig. 1:**
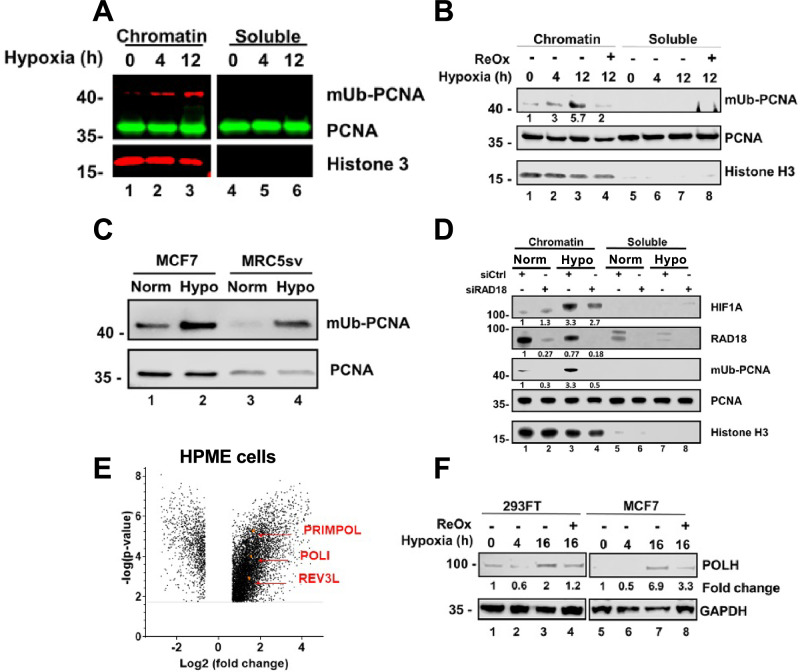
Hypoxia triggers PCNA monoubiquitination and increases expression of TLS DNA polymerases. **A** HEK293FT cells were subjected to hypoxia (0.5% Oxygen) for 4 or 12 h. Chromatin-bound and soluble proteins were extracted, and analyzed by immunoblotting after running it on 4–20% ExpressPlus™ PAGE gel (Genscript) in SDS-MOPS buffer. **B** As in A, except that the cells were also subjected to hypoxia for 12 h, followed by 1 h of reoxygenation. **C** MCF7 and MRC5sv cells were subjected to 24 h of hypoxia (0.5% oxygen), after which total cell proteins were fractionated and immunoblotted for PCNA and mUb-PCNA. **D** HEK293FT cells were treated with siRNA against human RAD18 (50 nM) or its non-targeting control for 48 h and then subjected to hypoxia (0.5% oxygen) for 24 h after which its chromatin-bound and soluble proteins were analyzed by immunblotting for the presence of the indicated proteins, as described for panels **A** and **B**. **E** Expression data obtained from a free access data source, analyzed using Genevestigator software. Effect size threshold was 1.5-fold change (0.6 in log2 scale), and FDR threshold was 0.05 (all had an FDR score under 0.01, and almost all under 0.001). A Human Pulmonary microvascular endothelium (HPME) cell line was subjected to hypoxia (1% oxygen for 48 h; two samples) compared to untreated samples (three samples in normoxia). The effect of hypoxia on *POLI*, *REV3L* and *PRIMPOL* is highlighted. **F** HEK293FT and MCF7 cells were subjected to hypoxia with and without reoxygenation of 1 h, and analyzed by Western blot for the presence of DNA polymerase η.

A key regulatory mechanism of TLS is the monoubiquitination of PCNA [16, 17], which function in the recruitment of TLS DNA polymerases to the fork [16, 18–20]. To examine the effect of hypoxia on PCNA monoubiquitination, we exposed HEK293FT cells to hypoxia (0.5% oxygen) and analyzed PCNA using Western blotting. As can be seen in Fig. 1A, we found that hypoxia induces PCNA monoubiquitination. Reoxygenation for one hour, after 12 h of hypoxia, reduced the effect (Fig. 1B). The PCNA mono-ubiquitination was also observed in two other human cell lines: MRC5sv, a human SV40-transformed lung fibroblast cell line, and the breast cancer MCF7 cell line (Fig. 1C). Knocking-down the expression of RAD18, the canonical E3-ligase that ubiquitinates PCNA [18], strongly reduced PCNA monoubiquitination under hypoxia (Fig. 1D), indicating that Rad18 is responsible also for the increased monoubiquitination under hypoxia.

Transcriptome analysis, from an online free-access database of the human pulmonary microvascular endothelium (HPME) cell line subjected to hypoxia (1% oxygen for 48 h; two samples), revealed that the expression of the genes encoding DNA polymerase ι (*POLI*), the catalytic subunit of DNA polymerase ζ (*REV3L*), and PrimPol (*PRIMPOL*), each increased by more than 2.5-fold (Q-value < 0.001) compared to untreated samples (three samples under normoxia) (Fig. 1E). We next checked the protein level of DNA polymerase η (POLH) in HEK293FT cells under hypoxia, and found that the protein level of POLH was increased after 16 h of hypoxia, and then suppressed after 1 h of reoxygenation (Fig. 1F). Similar effects were observed in MCF7 cells (Fig. 1F). Thus, the amounts of POLH increased under hypoxia, and based on the mRNA expression this may be true for additional TLS DNA polymerases.

### The HIF1 pathway regulates PCNA monoubiquitination and expression of TLS DNA polymerases

**Fig. 2:**
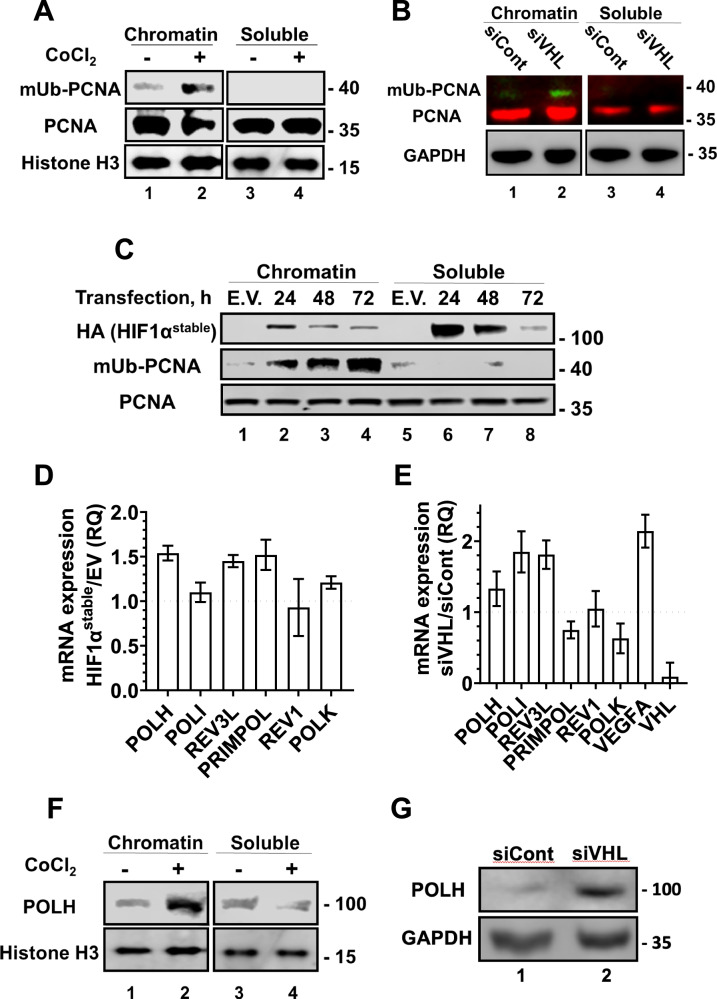
The HIF1 pathway induces PCNA mono-ubiquitination and expression of TLS polymerases. Cell lines were subjected to various treatments that cause HIF1α accumulation **A–C**, after which their protein content was separated into chromatin-bound and soluble fractions, and fractionated by SDS-PAGE followed by immune-blotting with the indicated antibodies. **A** HEK293FT cells treated with 100 µM CoCl_2_ for 24 h. **B** MCF7 cells transfected with siRNA against human *VHL* (50 nM) for 48 h. **C** MCF7 cells were transfected with the HA-HIF**1**α ^P402A/P564A^ plasmid or an empty vector (E.V.) for the indicated time periods. For E.V. the 24 h time point is shown, with longer times having similar results. **D** HEK293FT cells were transfected with HIF1A^P402A/P564A^ for 24 h and then analyzed using qPCR. **E** MCF7 cells were treated with siRNA against *VHL* gene for 72 h and then analyzed using qPCR. **F** HEK293FT cells were treated with 100 µM CoCl_2_ for 24 h, or **G** with 50 nM siRNA against the *VHL* gene for 72 h, followed by SDS-PAGE and Western blot analysis for the indicated proteins.

To examine whether the effect of hypoxia on TLS is regulated by the PHD-VHL-HIF1 pathway, we used three methods, each leading to the accumulation of HIF1α, a key hypoxia regulator: CoCl_2_ treatment, which suppresses PHD activity [21]; siRNA knockdown of the *VHL* gene, and Transfection of HEK293FT cells with HA-HIF1α^P402A/P564A^, an HA-tagged HIF1α variant with mutations that make it stable under normoxia conditions. We found that each of the treatments caused increased PCNA monoubiquitination (Fig. 2A–C). Thus, monoubiquitination of PCNA under hypoxia appears to be regulated by the HIF1 pathway.

RT-qPCR analysis of RNA extracted from HEK293FT cells expressing HIF1A^P402A/P564A^ and grown under normoxia revealed increased expression of about 50% of the *POLH*, *REV3L* and *PRIMPOL* TLS DNA polymerases genes (Fig. 2D), and a generally similar effect was observed by knocking-down the expression of *VHL* (Fig. 2E). Although these effects are modest, they are reproducible. To examine the effect of HIF1α on TLS DNA polymerases at the protein level, we treated HEK293FT with CoCl_2_ or si*VHL* and found that in both cases the amount of the POLH protein increased (Fig. 2F, G). Thus, the effect of hypoxia on TLS proteins appears to be mediated largely by the HIF1 pathway.

**Fig. 3:**
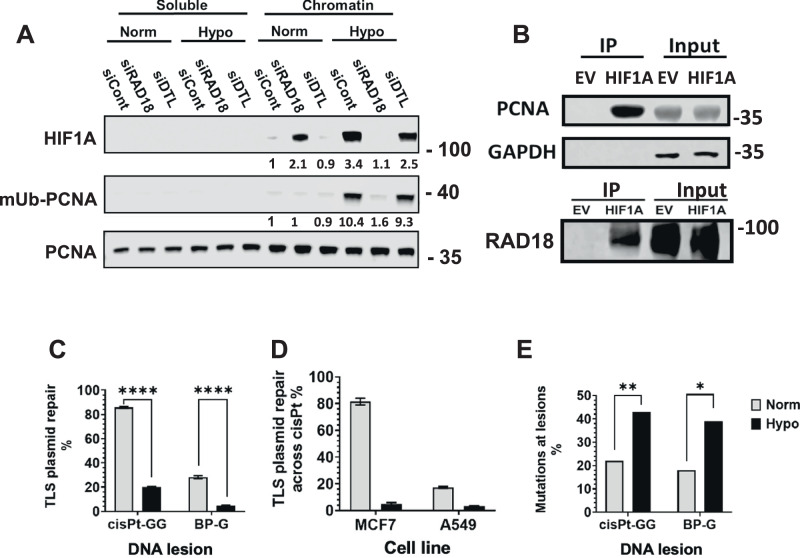
Analysis of HIF1α interactions and TLS across DNA lesions under hypoxia. **A** HEK293FT cells were transfected with the indicated siRNA (50 nM) and incubated for 48 h, followed by 24 h incubation under hypoxia (0.5% oxygen) after which chromatin-bound and soluble proteins **A** were analyzed by immunoblotting for the presence of the indicated proteins, as described above. **B** HEK293FT cells were transfected with HA-HIF**1**α^P402A/P564A^ expressing plasmid or the empty vector for 24 h. Proteins were then extracted and immunoprecipitated (20μg) with an anti-HA antibody, followed by SDS-PAGE and blotting with the indicated antibodies. **C**–**E** Cells were subjected to hypoxia or normoxia for 24 h after which they were assayed for TLS activity using the TLS gap-lesion plasmid assay. **C** The effect of hypoxia on TLS across a cisPt-GG or BP-G lesion in HEK293FT cells. **D** The effect of hypoxia on TLS across a cisPt-GG lesion in MCF7 (breast cancer) and A549 (lung cancer) cells. **E** TLS mutagenicity under hypoxia was assessed by sequencing the region filled-in by TLS in gap-lesion plasmids with a cisPt-GG or BP-G adduct. The detailed sequence data is presented in Supplementary Table S1.

To gain an indication on the mode of involvement of HIF1α in PCNA ubiquitination we examined the level of HIF1α in cells in which RAD18 expression was knocked down. As can be seen in Fig. 3A, cells under hypoxia treated with control siRNA, showed PCNA ubiquitination as well as the presence of HIF1α, consistent with the results in Fig. 1D. Treatment with siRAD18 led to a strong decrease in PCNA monoubiquitination, as expected. Interestingly, it also led to a strong decrease in the amount of HIF1α (Fig. 3A). Treatment under hypoxia with siDTL, which targets a non-canonical E3 ligase that can ubiquitinate PCNA, had marginal, if any, effect on monoubiquitinated PCNA and HIF1α (Fig. 3A). Under normoxia HIF1α was not observed, except under treatment with siRAD18, the reasons for which are not clear yet. To examine the possibility of an interaction between HIF1α and RAD18, we transfected cells with HA-HIF1α^P402A/P564A^ or a control empty vector, and immunoprecipitated the HIF1α using an anti-HA antibody. As can be seen in Fig. 3B, PCNA as well as Rad18 were immunoprecipitated in cells transfected with HA-HIF1α^P402A/P564A^, but not with the empty vector. Under both conditions GAPDH, used as a control, was not immunoprecipitated. Taken together these results hint that the stimulation of PCNA mono-ubiquitination by HIF1α may be mediated via its interaction with RAD18 and PCNA.

### Lesion bypass is reduced under hypoxia, with residual bypass becoming more mutagenic

To examine whether lesion bypass is functionally affected by hypoxia, we used a TLS assay based on a gapped plasmid, carrying a specific lesion in the ssDNA region, which was previously shown to be effective for studying TLS (e.g., [22–24]). We used a plasmid with either an intrastrand guanine-guanine adduct (cisPt-GG) formed by the chemotherapy drug cisplatin, or a (+)-*trans*-BPDE-N2-dG (BP-G) adduct, known to be formed in DNA by tobacco smoke. Exposure of human embryonic kidney HEK293FT cells to 16 h of acute hypoxic conditions (0.5% oxygen), followed by transfection with the gap-lesion plasmids and continued incubation under hypoxia, decreased the extent of lesion bypass by 4–5 -fold for either the cisPt-GG or BP-G lesions (Fig. 3C). Similar effects were observed with two additional cell lines, the breast cancer cell line MCF7 and the lung cancer cell line A549 (Fig. 3D). Thus, while PCNA monoubiquitination and expression of TLS DNA polymerases increased under hypoxia, contrary to our expectation, TLS was reduced.

DNA sequence analysis of the regions opposite to the lesion revealed that for each lesion the fidelity decreased during hypoxia, with a twofold rise in mutagenicity compared to normoxia (Fig. 3E and Supplementary Table S1). Thus, TLS lesion bypass is suppressed under hypoxia, with the residual lesion bypass becoming more mutagenic.

### TLS DNA polymerases are required for effective DNA replication and maintaining cell viability under hypoxia

The decrease in TLS lesion bypass activity under hypoxia, despite the increase in TLS DNA polymerases and PCNA monoubiquitination, prompted us to consider whether the TLS low-fidelity DNA polymerases are involved in another process under hypoxic conditions. Because several reports presented evidence that TLS DNA polymerases assist genome replication through difficult-to-replicate regions [25–28], we investigated the possibility of specific involvement of TLS DNA polymerases in global genomic replication under hypoxia.

**Fig. 4:**
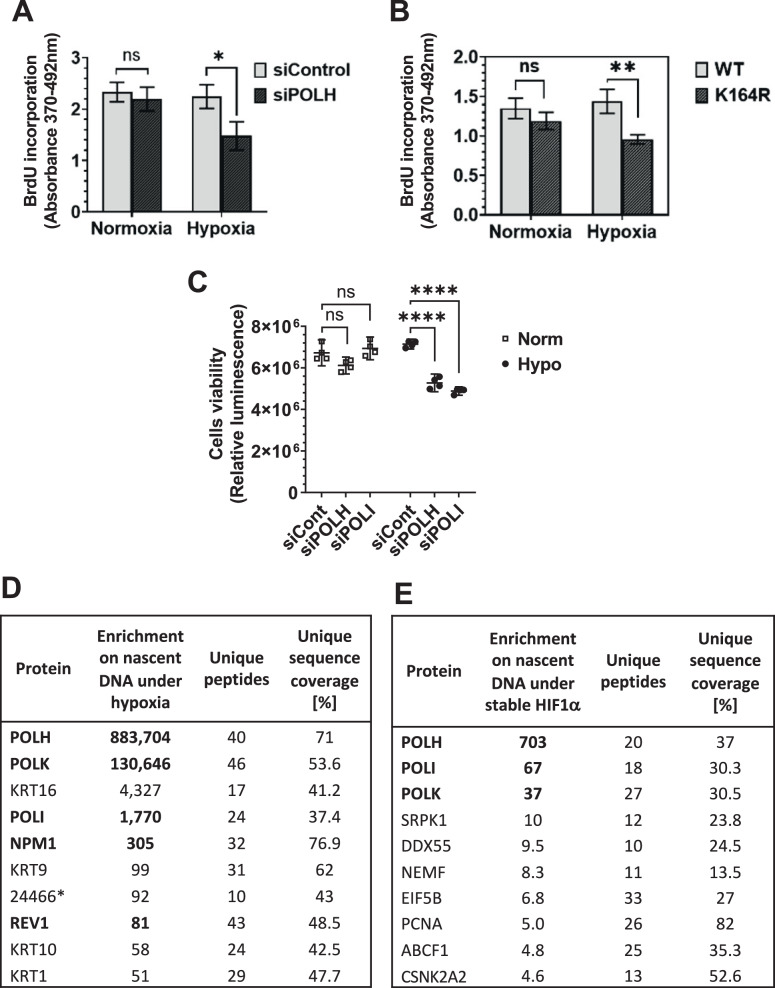
TLS is involved in global DNA replication during hypoxia. **A** MCF7 cells were transfected with the indicated siRNA (50 nM) and incubated for 48 h. After that, the cells were exposed to hypoxia (0.5% oxygen) for 16 h, treated with BrdU for two hours, and fixated with ethanol. BrdU incorporation was analyzed using Roche’s ELISA, BrdU (colorimetric) kit according to manufacturer’s protocol. **B** MEFs with either a *Pcna-K164R* mutation or WT PCNA, were grown under hypoxia, and BrdU incorporation was measured as described in **A**. **C** XP12RO human cells were treated with the indicated siRNAs, followed by hypoxia and then viability analysis using the CellTiter-Glo® Luminescence Assay. **D**,**E** HEK293FT cells were subjected to 16 h hypoxia (0.5% oxygen) **D** or transfected with HA-HIF1A^P402A/P564A^ under normoxia **E**, and the iPOND protocol was then conducted. In short, the cells were supplemented with EdU or DMSO, and after 60 min were crosslinked, permeabilized, and biotin was added for a click reaction with the EdU. The cells were lysed, and Streptavidin beads were added to capture the biotin. After a wash, the beads were boiled in an elution buffer, and the samples were analyzed by mass spectrometry. Hypoxia-specific enrichments of proteins on nascent DNA **D** was calculated from the MS results (Data Set 1) as: (Hypoxia Pulse/Chase LFQ-intensity ratio)/(Normoxia Pulse/Chase LFQ-intensity ratio). HIF1α-specific enrichments of proteins on nascent DNA **E** was calculated from the MS results (Data Set 2) as: (HIF1α Pulse/Chase LFQ-intensity ratio)/(Empty Vector Pulse/Chase LFQ-intensity ratio). The two datasets were deposited in the Figshare Repository at 10.6084/m9.figshare.27061339. The lists were filtered for proteins identified based on ≥10 unique peptides with ≥10% unique sequence coverage, and ranked in descending protein enrichment ratio. The 10 highest ranking proteins enriched on nascent DNA under hypoxia **D** or in normoxic cells expressing stable HIF1α **E** are presented.

To that end we knocked down the expression of *POLH* in MCF7 cells, and measured the incorporation of bromodeoxyuridine (BrdU; a thymidine analog) under hypoxia compared to normoxia. No significant effect on BrdU incorporation was observed under normoxia for cells in which *POLH* was knocked down compared to control siRNA treatment. In contrast, during hypoxia, cells in which *POLH* expression was knocked down exhibited lower BrdU incorporation than cells treated with siRNA control (Fig. 4A), suggesting that DNA polymerase η plays a much more significant role in replication during hypoxia than during normoxia.

We next examined BrdU incorporation under hypoxia of immortalized mouse embryonic fibroblasts (MEF) cells with a *Pcna*^*K164R/K164R*^ mutation, which does not allow PCNA-K164 ubiquitination, thereby weakening PCNA-TLS polymerases interaction. The PCNA-K164R mutant cells exhibited lower BrdU incorporation than WT (*Pcna*^*+/+*^) cells (Fig. 4B), further supporting the notion that TLS, and specifically mUb-PCNA, have a more significant role in replication under hypoxia than under normoxia.

An involvement in global DNA replication under hypoxia might be reflected in cell viability. To examine this possibility, we knocked down TLS DNA polymerases using siRNA in human XP12RO cells incubated under hypoxic conditions (0.5% oxygen for 24 h) or normoxic conditions. The cell line used is from an XPA patient, whose nucleotide excision repair (NER) is deficient, making TLS more important for maintaining genomic stability. As can be seen in Fig. 4C, knockdown of *POLI* or *POLH* each decreased cell viability under hypoxia by 31% and 29% respectively (*P*-value < 0.0001). In contrast, under normoxia, knocking down each of these TLS DNA polymerases did not significantly affect cell viability (Fig. 4C). A similar experiment performed with a NER-proficient cell line did not show an effect on viability (not shown). However, it should be pointed out that a considerable fraction of cancers exhibits somatic mutations in DNA repair genes, and therefore a systematic screen for such cancer cells may uncover a synthetic lethality of inhibiting TLS in a background of reduced repair. Of note, condition other than NER-deficiency may lead to elevated importance of TLS DNA polymerases in genomic replication. For example, the hypoxia induced oncogenic miR-155 is associated with decreased expression of the high-fidelity replicative DNA polymerase δ, likely making involvement of TLS DNA polymerases more important [29]. Thus, TLS DNA polymerases are important for maintaining cell viability under hypoxia, at least under NER-deficient conditions, consistent with a role in genomic DNA replication.

### TLS DNA polymerases are recruited to nascent DNA specifically under hypoxia

To further explore the involvement of TLS DNA polymerase in genomic DNA replication we used iPOND (isolation of Proteins On Nascent DNA), a method that enables the investigation of proteins at the replication fork [30]. In short, this method uses a pulse of EdU (an analog of thymidine), and after cross-link and wash, Streptavidin-Biotin click chemistry is used to pulldown the proteins from the nascent DNA. We grew HEK293FT cells under hypoxia (0.5% oxygen) or normoxia, performed iPOND, and used mass spectrometry to analyze the proteins. We found that there is massive recruitment of TLS DNA polymerases η, κ, ι and REV1 to nascent DNA (Fig. 4D) during hypoxia compared to normoxia. Thus, at least 4 low-fidelity TLS DNA polymerases are recruited to nascent DNA, specifically under hypoxic conditions.

We also examined whether HIF1 is involved in the recruitment of TLS DNA polymerases to nascent DNA, by performing iPOND analysis with cells expressing HA-HIF1α^P402A/P564A^ grown under normoxic conditions. Remarkably, the expression of the stable HIF1α was sufficient to cause a recruitment of TLS DNA polymerases η, κ, ι to nascent DNA compared to cells expressing a control empty vector (Fig. 4E).

### HIF1 pathway activation and TLS genes mRNA expression correlate in renal cell carcinoma tumors

Tumors from 80% of clear cell renal cell carcinoma (ccRCC) patients have mutations in the *VHL* gene [31]. This enables us to examine the relationship between our results obtained with cell cultures, to the in vivo situation, by analyzing the correlation between mRNA expression of TLS polymerase genes and the *VEGFA* gene, a known target of HIF1α. We found positive correlations between the expression of *VEGFA*, and *POLH*, *POLI*, and *REV3L* - 0.45, 0.49, and 0.36, respectively. When compared the top 20% of *VEGFA* expressing tumor samples to the bottom 20% expressing tumors and found that *POLH* goes up 1.69-fold (FDR 1 × 10^−10^), *POLI* goes up 2.06-fold (FDR 1.3 × 10^−15^) and *REV3L* goes up 1.62-fold (FDR 3.8 × 10^−6^; Supplementary Table S2). Although preliminary and only correlative, these results suggest that HIF1α may be involved in induction of error-prone DNA polymerases in a human cancer.

## Discussion

While TLS functions primarily to overcome replication obstacles, mainly DNA lesions, our results indicate that under hypoxia TLS proteins are induced, and assume an important role in genome replication. What function might this fulfill? While hypoxia does not appear to cause base damage [32], which is typically bypassed by TLS, it does causes replication stress, namely difficulties in replication caused by a variety of reasons, e.g., decreased expression of essential replication proteins, shortage in nucleotides, etc., [33, 34] which may be alleviated, in part, by TLS error-prone DNA polymerases. Moreover, it is possible that hypoxia triggers changes in the genome structure, such that a bigger part of the genome becomes difficult-to-replicate, requiring the assistance of error-prone DNA polymerases. Indeed, it was reported that hypoxia increased H3K9me3 and H3K9me2, histone modifications associated with heterochromatin [34, 35]. Since the involvement of TLS replication in difficult-to-replicate regions of the genome, such as fragile sites [25, 26, 36] and heterochromatin [28], was already reported, it is possible that the involvement of TLS DNA polymerases in genomic replication under hypoxia functions to enable replication of vast regions of difficult-to-replicate regions, such as heterochromatin. However, further experimentation is needed to explore this, as well as other possible explanations. While the detailed mechanism of the involvement of TLS in genomic replication during hypoxia is yet to be uncovered, the results described above reveal the involvement of HIF1α, which for PCNA ubiquitination, is likely facilitated via interaction of HIF1α with RAD18 and PCNA.

Our results demonstrating massive recruitment of TLS DNA polymerases to genomic replication under hypoxia adds a new non-canonical function to the list of TLS functions other than the canonical Trans-Lesion DNA Synthesis, namely somatic hypermutation in the immune system [37] and replication of difficult to replicate (e.g., heterochromatin and fragile DNA sites) in the genome [25, 28, 36]. It is consistent with the report that error-prone DNA polymerases are attracted to gaps under hypoxia, leading to increased mutations [38]. Thus, we suggest that TLS should be regarded not only as a lesion-bypassing mechanism (lesion-bypass-TLS) only, but also as a ‘rough-terrain’ replicative process (replicative-TLS). This may require a slightly different TLS machine, perhaps with different auxiliary proteins or proteins modifications than the lesion-bypass TLS machines. Importantly, this increase in non-canonical TLS activity suggest that the tumor microenvironment leads the cell to forgo, to some part, the fast and accurate canonical DNA polymerases, for the more flexible and robust, but sloppy TLS polymerases. By doing that the cancer cells possibly gain the durability to help cope with replication stress, and the diversification, to escape the body’s immune system and drugs [38].

TLS DNA polymerases are attractive targets for cancer therapy, because inactivating them is expected to inhibit the development of drug resistance, e.g., cisplatin-induced drug resistance [39], without increasing drug-induced mutagenesis [40]. Their important role in genome replication under hypoxia further strengthens their attractiveness as potential useful targets for restraining drug resistance.

## Materials and methods

**Cells culture, transfection, Protein and RNA extraction, Western blotting, RT-qPCR and LC-MS/MS analysis** are described in the Supplementary Information.

### Hypoxia treatment

Either an active hypoxia chamber (COY, O_2_ Control InVitro Cabinet; courtesy of Prof. Gad Asher) or passive hypoxia box (in-house) were used to create hypoxic (0.5% O_2_) with 5% CO_2_ and were inserted to 37 °C incubators to maintain suitable temperature. Cells were harvested using scraper and cold PBS and frozen in liquid N_2_, spending no more than 5–10 min under normoxia. CoCl_2_ treatment was 100 µM for 24 h unless mentioned otherwise.

### TLS assay

The TLS gap lesion plasmid assay was previously described [22–24], and is briefly described in Supplementary Information.

### BrdU incorporation assay

The Cell Proliferation ELISA, BrdU (colorimetric) kit (Merck) was used for measuring BrdU incorporation. 5000 cells were seeded per well in a 96-wells plate, a day in advance. BrdU was added to each well, and after two hours of incubation the cells were fixed, and BrdU incorporation determined following the kit’s instructions. Absorbance was measured at 370 nm using a plate reader (Tecan).

### Cell viability

Cell viability was measured using CellTiter Glo® Luminescent Cell Viability Assay (Promega) according to the manufacturer.

### Isolation of protein on nascent DNA (iPOND)

To analyze the proteins on nascent DNA the iPOND method was performed as described [30], and briefly described in the Supplementary Information.

## Supplementary information

Supplemental Information

## Data Availability

All data generated or analyzed during this study are included in this published article and its supplementary information files. The datasets of proteins present on nascent DNA in the iPOND experiments was deposited in the Figshare Repository at 10.6084/m9.figshare.27061339.
