# Supplementary material for: Hypoxia-dependent recruitment of error-prone DNA polymerases to genome replication
Source: Oncogene. 2024 Oct 28;44(1):42–9. doi: 10.1038/s41388-024-03192-0 (PMC11700837; doi:10.1038/s41388-024-03192-0)
Supplement: Supplementary file 1 — Supplemental Information [file 41388_2024_3192_MOESM1_ESM.pdf]

Supplementary Information

Hypoxia-Dependent Recruitment of Error-Prone DNA Polymerases to Genome Replication

Ran Yehuda[1], Ido Dromi[1], Yishai Levin[2], Thomas Carell[3], Nicholas Geacintov[4] and Zvi Livneh[1*]

[1] Dept. of Biomolecular Sciences, Weizmann Institute of Science, Rehovot, 7610001, Israel
[2] The de Botton Institute for Protein Profiling of the Nancy and Stephen Grand Israel National Center for Personalized Medicine, Weizmann Institute of Science, Rehovot, 7610001, Israel
[3] Center for Integrated Protein Science at the Department of Chemistry, Ludwig-Maximilians-Universität, München, Butenandtstrasse 5-13, 81377, München, Germany
[4] Chemistry Department, New York University, New York, NY, USA

Supplementary Methods

Cells culture and transfection

Human fibroblasts XPA (XP12RO; xeroderma pigmentosum patient cells) and SV40-transformed lung fibroblast MRC5sv (gifts from A. R. Lehmann, University of Sussex, Brighton, UK), lung cancer A549 cells, Human embryonic kidney HEK293FT cells, and breast cancer MCF7 cells, were each cultured in Dulbecco's modified Eagle's medium (DMEM; Gibco) supplemented with 10% fetal bovine serum (FBS; Hyclone), and 100 µg/ml of streptomycin, 100 units/ml of penicillin, 2 mM L-Alanyl-L-Glutamine, 1 mM sodium pyruvate (Biological Industries). Chinese hamster ovaries CHO-K1 were maintained in DMEM-F12 (Gibco) supplemented with 10% FBS (Hyclone), and 100 µg/ml of streptomycin, 100 units/ml of penicillin (Biological Industries).

For knockdown with siRNA, RNAiMAX (Invitrogen) transfection reagent was used with either 25nM or 50nM siRNA (Dharmacon, siGENOME SMART pool and ON-TARGETplus SMART pool; Dharmacon) for 48 or 72 hours. Transfection was conducted according to protocol using lipofectamine 2000 (Invitrogen) in gap lesion plasmid assay, or JetPRIME (Polyplus) for protein expression.

Protein extraction and Western blotting

For whole cell protein extraction, CelLytic-M lysis buffer (Sigma-Aldrich) was used supplemented with 2.5mM $MgCl_2$, 50 units/ml Benzonase, protease inhibitors cocktail, and phosphatase inhibitors. Samples with the lysis buffer were incubated for 30 min at room temperature. For preparing chromatin-bound and soluble protein fractions, the protocol described in (1) was used. 30µg protein were loaded on 4-20% ExpressPlus™ PAGE gel (Genscript) and ran in SDS-MOPS

buffer. Primary antibodies used were PC10 (Santa Cruz, sc-56), and antibodies against ubiquityl-PCNA (Lys164; Cell Signaling, 13439S), Histone H3 (Cell signaling, 9715S), RAD18 (Cell Signaling, 9040S), GAPDH (Cell Signaling, 5174S), POLH Antibody (B-7; Santa Cruz, sc-17770), and HIF1A (D1S7W; Cell Signaling, 36169S).

<u>RNA purification and qPCR analysis</u>
Purification of RNA was done using RNeasy plus mini kit (Qiagen), and included treatment with RNase-free DNase I (Qiagen), followed by cDNA synthesis using the High Capacity cDNA Reverse Transcription Kit (Applied Biosystems). Primers were predesigned using KiCqStart SYBR® Green primers (Sigma-Aldrich). qPCR was performed using KAPA SYBR® FAST qPCR Master Mix (Sigma-Aldrich) and run on a QuantStudioTM 6 Flex Real-Time PCR System (Applied Biosystems). Each PCR reaction was done in quadruplicate, and the analysis was done using ΔΔCt method.

<u>TLS assay</u>
The TLS gap lesion plasmid assay was previously described (2-5). Briefly, cells were co-transfected using lipofectamine 2000 (Invitrogen) with plasmids containing a ssDNA gap, either with a lesion (kanamycin-resistant; Kan^R) or no lesion (chroramphenicol-resistant; Cm^R). After incubation of 5-16h the plasmid content was extracted, electroporated into TLS-defective *E. coli recA* strain JM109, and seeded on LB-Cm and LB-Kan agar plates. The ratio between the numbers of colonies grown on the LB-Kan (with the lesion) to LB-Cm (control) gives the gap repair efficiency. To analyze the TLS extent and mutagenicity, colonies from the LB-Kan plates were picked and the filled gap region was sequenced. To measure TLS under hypoxia, cells were incubated under hypoxic conditions for 16h before transfection, then transfected with the gapped plasmids under normoxia (2 min), followed by an additional incubation of 6-8 hours under hypoxia. Harvesting and all subsequent were done under normoxia. Each TLS experiment was performed 3-4 times.

<u>Isolation of Protein On Nascent DNA (iPOND)</u>
The iPOND method was performed as described (6). In short, after HEK293FT were exposed to 16h hypoxia (<0.5%), 10μM 5-ethynyl-2'-deoxyuridine (EdU) was added for 45 min flowed by 20min crosslink with 1% formaldehyde. For controls, one sample was not supplemented with EdU and in another sample 60 min or thymidine chase was done before crosslink. The cells were then washed, permeabilized and washed again before the addition of click reaction buffer (10 mM sodium ascorbate, 2 mM $CuSO_4$, 10 μM biotin-azide, in PBS) for 2 hours. The cells were then washed and sonicate in lysis buffer (1% SDS in 50 mM Tris-HCl) and centrifuge. The supernatant

was collected and 15µl were saved as input for later analysis. Magnetic-Streptavidin beads (Dynabeads™ M-280 Streptavidin, Invitrogen™, thermofisher scientific) was added and the samples were rotated in 4°C overnight. The beads were washed, and then boiled for 25 min in elution buffer (5% SDS, 50 mM Tris-HCl, pH 7.5). Samples were stored in -80°C until mass spectrometry (MS) analysis.

LC-MS/MS analysis

Each sample was loaded using split-less nano-Ultra Performance Liquid Chromatography (nanoAcquity; Waters, Milford, MA, USA). The mobile phase was: A) H_2O + 0.1% formic acid and B) acetonitrile + 0.1% formic acid. Desalting of the samples was performed online using a reversed-phase Symmetry C18 trapping column (180 µm internal diameter, 20 mm length, 5 µm particle size; Waters). The peptides were then separated using a T3 HSS nano-column (75 µm internal diameter, 250 mm length, 1.8 µm particle size; Waters) at 0.35 µL/min. Peptides were eluted from the column into the mass spectrometer using the following gradient: 4% to 30%B in 155 min, 35% to 90%B in 5 min, maintained at 90% for 5 min and then back to initial conditions.

The nanoUPLC was coupled online through a nanoESI emitter (10 µm tip; FossilIonTech, Spain) to a quadrupole orbitrap mass spectrometer (Q Exactive Plus, Thermo Scientific).

Data was acquired in data dependent acquisition (DDA) mode, using a Top10 method. MS1 resolution was set to 70,000 (at 400m/z), mass range of 375-1650m/z, AGC of 1e6 and maximum injection time was set to 60msec. MS2 resolution was set to 17,500, quadrupole isolation 1.7m/z, AGC of 1e5, dynamic exclusion of 45sec and maximum injection time of 60msec.

Raw data was processed with MaxQuant version 1.6.6.0 with the default parameters and modifications. Sequence database search was done against the human sequences from UniprotKB version 11_2019. Match between runs was enabled.

The datasets of proteins present on nascent DNA in the iPOND experiments (Dataset 1 and Dataset 2) were deposited in the Figshare Repository at https://doi.org/10.6084/m9.figshare.27061339.

Bioinformatics

TCGA expression data was downloaded via Xena cancer browser, and filtered to keep only primary tumors. Mutation annotation files (maf) were downloaded via Firebrowse. Pearson correlation was tested between VEGFA and the TLS selected genes in each of the selected cancer types. For each gene, the samples with the top 20% of VEGFA expression were compared

with the samples with the bottom 20% of VEGFA expression with a t-test. FDR was used for multiple-test correction.

1. Hendel A, Krijger PH, Diamant N, Goren Z, Langerak P, Kim J, et al. PCNA ubiquitination is important, but not essential for translesion DNA synthesis in mammalian cells. PLoS Genet. 2011;7(9):e1002262.
2. Diamant N, Hendel A, Vered I, Carell T, Reissner T, de Wind N, et al. DNA damage bypass operates in the S and G2 phases of the cell cycle and exhibits differential mutagenicity. Nucleic Acids Res. 2012;40:170-80.
3. Shachar S, Ziv O, Avkin S, Adar S, Wittschieben J, Reissner T, et al. Two-polymerase mechanisms dictate error-free and error-prone translesion DNA synthesis in mammals. EMBO J. 2009;28(4):383-93.
4. Ziv O, Zeisel A, Mirlas-Neisberg N, Swain U, Nevo R, Ben-Chetrit N, et al. Identification of novel DNA-damage tolerance genes reveals regulation of translesion DNA synthesis by nucleophosmin. Nature Commun. 2014;5:5437.
5. Ziv O, Diamant N, Shachar S, Hendel A, Livneh Z. Quantitative measurement of translesion DNA synthesis in mammalian cells. Methods Mol Biol. 2012;920:529-42.
6. Sirbu BM, Couch FB, Cortez D. Monitoring the spatiotemporal dynamics of proteins at replication forks and in assembled chromatin using isolation of proteins on nascent DNA. Nat Protoc. 2012;7(3):594-605.

Table S1: Sequencing results of the DNA region opposite to cisplatin-GG DNA lesion of cells treated with hypoxia or normoxia obtained using TLS assay

Treatment:<br>DNA damage	Normoxia	Hypoxia
**cisPt-GG** (5' A-GG-C '3)<br>Nucleotide inserted opposite lesion<br>(5' → '3)	Number of isolates (%)	
G-CC-T	70/95 (74)	47/92 (51)
G-C**A**-T	16/95 (17)	32/92 (35)
G-C**T**-T	3/95 (3)	2/92 (2)
G-**TT**-T	-	1/92 (1)
G-**AA**-T	1/95 (1)	-
Non-TLS events	5 (5)	10 (11)
Total clones	95	92
Total TLS events analyzed	90 (100)	82 (100)
Accurate TLS frequency (%)	70/90 (78)	47/82 (57)
Mutagenic TLS (%)	20/90 (22)	35/82 (43)
Chi-square test (P-value)	0.004	

**BP-G** (5' T-G-C '3)<br>Nucleotide inserted opposite lesion<br>(5' → '3)		
T-G-C	37/45 (82)	28/46 (61)
T-T-C	8/45 (18)	16/46 (35)
T-C-C	-	2/46 (4)
Non-TLS events	0 (0)	0 (0)
Total clones	45	46
Total TLS events analyzed	45 (100)	46 (100)
Accurate TLS frequency (%)	37/45 (82)	28/46 (61)
Mutagenic TLS (%)	8/45 (18)	18/46 (39)
Chi-square test (P-value)	0.02	

HEK293FT cells were subjected to hypoxia or normoxia, transfected with cisPt-GG gap lesion plasmid, and then analyzed using TLS assay as described above.

Table S2: Comparison of the expression of TLS DNA polymerase genes among the top 20% of *VEGFA* expressing tumor samples to the bottom 20% expressing tumors

	Gene	Fold change	FDR
TLS DNA polymerases	POLH	1.69	1.07E-10
	POLI	2.06	1.32E-15
	POLK	1.20	8.39E-02
	REV1	1.50	5.76E-11
	REV3L	1.62	3.82E-06
	PRIMPOL	1.23	2.82E-03
	REV7	0.81	5.26E-02
House-keeping genes	ACTB	0.77	3.15E-05
	RPS18	1.20	6.34E-02
	B2M	0.91	2.76E-01
	AHSP	1.25	2.93E-01
